# The expendables: Bioarchaeological evidence for pauper apprentices in 19^th^ century England and the health consequences of child labour

**DOI:** 10.1371/journal.pone.0284970

**Published:** 2023-05-17

**Authors:** Rebecca L. Gowland, Anwen C. Caffell, Leslie Quade, Alysa Levene, Andrew R. Millard, Malin Holst, Poppy Yapp, S. Delaney, Chloe Brown, Geoff Nowell, Colin McPherson, Heidi A. Shaw, Nicolas A. Stewart, Sally Robinson, Janet Montgomery, Michelle M. Alexander

**Affiliations:** 1 Department of Archaeology, Durham University, Durham, United Kingdom; 2 Department of Anthropology, Masaryk University, Brno, Czech Republic; 3 School of History, Philosophy and Culture, Oxford Brookes University, Oxford, United Kingdom; 4 BioArCh, Department of Archaeology, University of York, York, United Kingdom; 5 BAAC, Hertogenbosch, The Netherlands; 6 Department of Earth Sciences, Durham University, Durham, United Kingdom; 7 School of Applied Sciences, University of Brighton, Brighton, United Kingdom; 8 Washburn Heritage Centre, Harrogate, North Yorkshire, United Kingdom; University of Otago, NEW ZEALAND

## Abstract

Child labour is the most common form of child abuse in the world today, with almost half of child workers employed in hazardous industries. The large-scale employment of children during the rapid industrialisation of the late 18^th^ and early 19^th^ centuries in England is well documented. During this period, the removal of pauper children from workhouses in cities to work as apprentices in rural mills in the North of England was commonplace. Whilst the experiences of some of these children have been recorded historically, this study provides the first direct evidence of their lives through bioarchaeological analysis. The excavation of a rural churchyard cemetery in the village of Fewston, North Yorkshire, yielded the skeletal remains of 154 individuals, including an unusually large proportion of children aged between 8 to 20 years. A multi-method approach was undertaken, including osteological and palaeopathological examination, stable isotope and amelogenin peptide analysis. The bioarchaeological results were integrated with historical data regarding a local textile mill in operation during the 18^th^-19^th^ centuries. The results for the children were compared to those obtained from contemporaneous individuals of known identity (from coffin plates) of comparable date. Most of the children exhibited distinctive ‘non-local’ isotope signatures and a diet low in animal protein when compared to the named local individuals. These children also showed severe growth delays and pathological lesions indicative of early life adversity, as well as respiratory disease, which is a known occupational hazard of mill work. This study has provided unique insights into the harrowing lives of these children; born into poverty and forced to work long hours in dangerous conditions. This analysis provides a stark testimony of the impacts of industrial labour on the health, growth and mortality risk of children, with implications for the present as well as our understanding of the past.

## Introduction

The industrial revolution transformed the economic and social landscape of Britain in the 18^th^ and 19^th^ centuries AD. The industrial boom was underpinned by the low-paid labour of women and children, which enabled British manufactories to be competitive in an increasingly globalized market [1]. In 1845, 43% of cotton workers were under 18 years of age and in other industries this figure was substantially higher [2]. Economic success came at a cost: historical documents and social commentators of the time paint a grim picture of the living and working conditions of the labouring class; characterized by low life expectancy, malnutrition, infection and occupational diseases [3, 4].

Much has been written about the rapid pace of industrialization during the late 18^th^ and 19^th^ centuries and the dramatic growth of manufacturing cities such as Manchester and Leeds, fuelled by economic migrants from the rural hinterlands [5]. Conditions within the cities were often dire, with poor quality housing, inadequate sanitation, heavily polluted air and water, and over-crowding [6]. While rural-to-urban migration was undoubtedly the most common direction of travel during this period, reverse economic migration–from town to country–also occurred. A prime example was the large number of pauper children who were removed from urban workhouses in cities such as London and Liverpool and sent to work in rural textile mills or farms, often far from home [7].

Parliamentary returns regarding the number of paupers in each parish for 1803 revealed that there were approximately 195,000 children aged from 5 to 14 years, in England and Wales, who required poor relief [8]. Consequently, workhouses located in the poorer parishes of cities were often overwhelmed by the destitute. During the same period, textile factories were being constructed in sparsely populated rural areas, close to watercourses (their source of power), but with a limited pool of local people to employ [9]. The transport of urban pauper children, often in ‘batches’, to work in these rural mills was a solution to both problems: it relieved urban parishes of the immediate financial and logistical burden of supporting poor children, whilst providing a ready source of cheap and malleable labour to these nascent industries [10]. Some London parishes even placed adverts in manufacturing areas to raise awareness of the availability of their children for work [7]. Boys and girls from as young as 7 years of age were apprenticed to low-skill trades in textile mills located in the North of England [11]. The large-scale employment of pauper apprentices during the late 18^th^ and early 19^th^ centuries was often essential to the economic viability of many textile businesses during their initial development [7]. These children would enter an apprenticeship by signing (or marking) an indenture, which was a contractual agreement binding them to their place of work until the age of 21 years (or marriage for girls) [12]. While some children signed up willingly, in many instances neither the children, nor their parents (if alive) had much agency in this arrangement, particularly if they were living in an over-crowded parish workhouse [7, 10]).

Parish apprentices were often transported many miles across the country for work; indeed, distance was desirable as it inhibited “the interference of idle or profligate relatives” (Liverpool Select Vestry as cited in Withall [13] (p. 214)). It also removed the children from the perceived “contagion” of poverty and its attendant associations of immorality and fecklessness, instead placing them on the path of becoming economically productive members of society. The apprenticeship of young children inevitably led in many instances to the break-up of family units, although occasionally siblings were indentured together [7].

The experiences of some pauper apprentices have been preserved via reports from government investigations into working conditions in factories and from autobiographical accounts (e.g. *The Memoir of Robert Blincoe* [14]). Historical research regarding the health and well-being of these children has yielded many important insights into their lives [7, 10, 13, 15, 16]. The extent to which child factory labour and apprenticeship was detrimental to the health of the children has, however, been the source of debate [17–19]. For example, mill work has been discussed as ‘light’ compared to agricultural labour [16], although the detrimental impact of cotton and flax dust on children’s lungs, as well as the risk of industrial accidents are acknowledged [16]. This study provides the first skeletal and biomolecular identification and analysis of pauper apprentices. The skeletal remains of these children were excavated from a cemetery located close to a textile mill in Fewston, North Yorkshire. Bioarchaeological evidence can resolve some of the existing debates by examining the direct impacts of living and working conditions, including occupational diseases such as respiratory conditions, on the growing bodies of children during this period. This study therefore yields, for the first time, direct and unique insights into the short lives of these children, and the effects of childhood and intergenerational poverty on growth and development.

It is important to note that this research has implications for the present as well as the past: the International Working Group on Child Labour has stated that “child labour is the single most common form of child abuse and neglect in the world today” [20 (p.539)]. Globally, nearly 1 in 10 children are subjected to child labour (over 160 million) and almost half of these are in work that is hazardous to their health and development [21]. Children also account for one quarter of enslaved people around the world today who, like the pauper apprentices, are often forced to work in gruelling and dangerous jobs. The toll on children’s health, growth and mortality today is not fully understood, rendering a study of past children, who endured similar working and living conditions to those today, all the more valuable.

### The Fewston churchyard: Skeletal assemblage and mill

**Fig 1:**
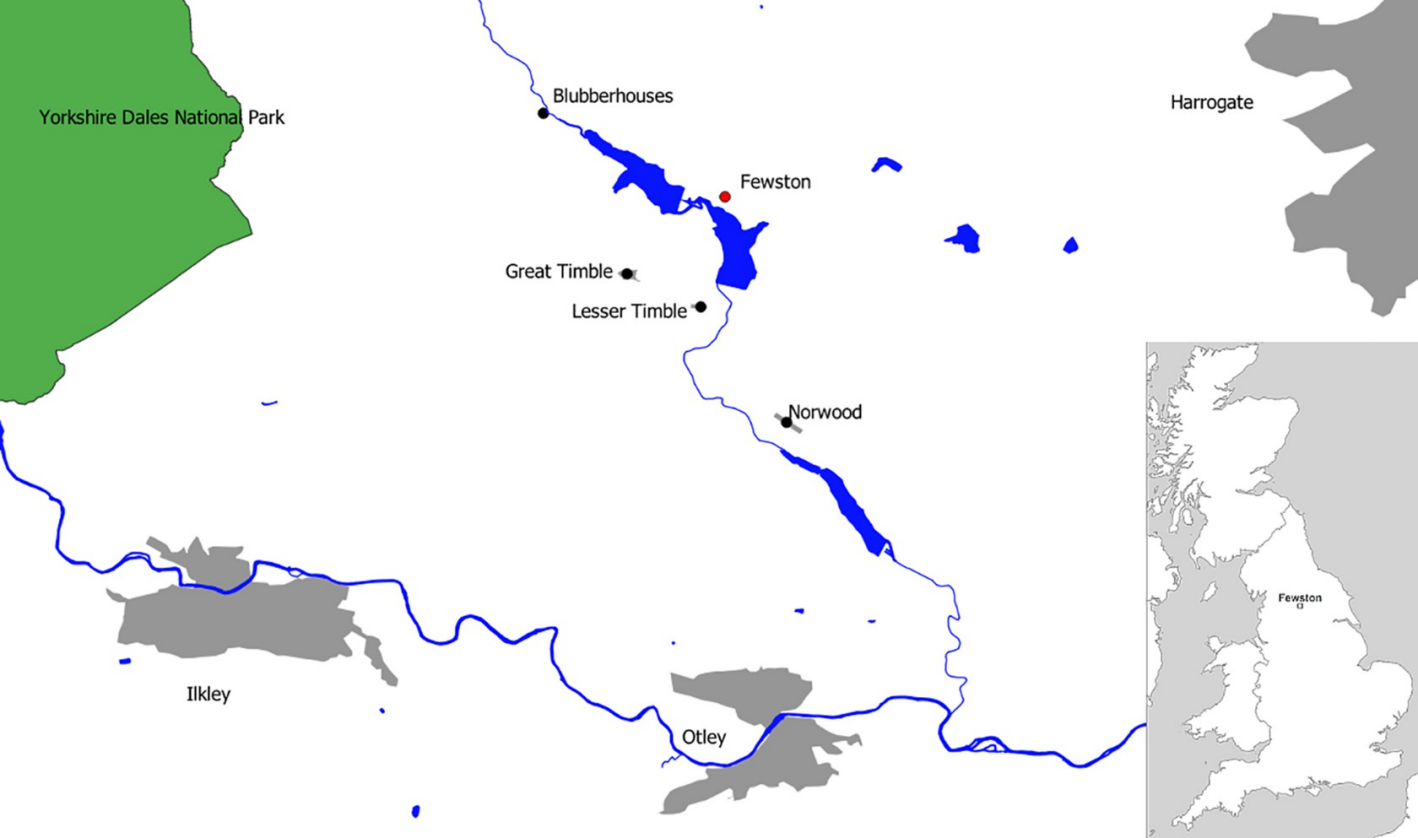
The location of the parish of Fewston; West House mill, located in Blubber houses.

**Fig 2:**
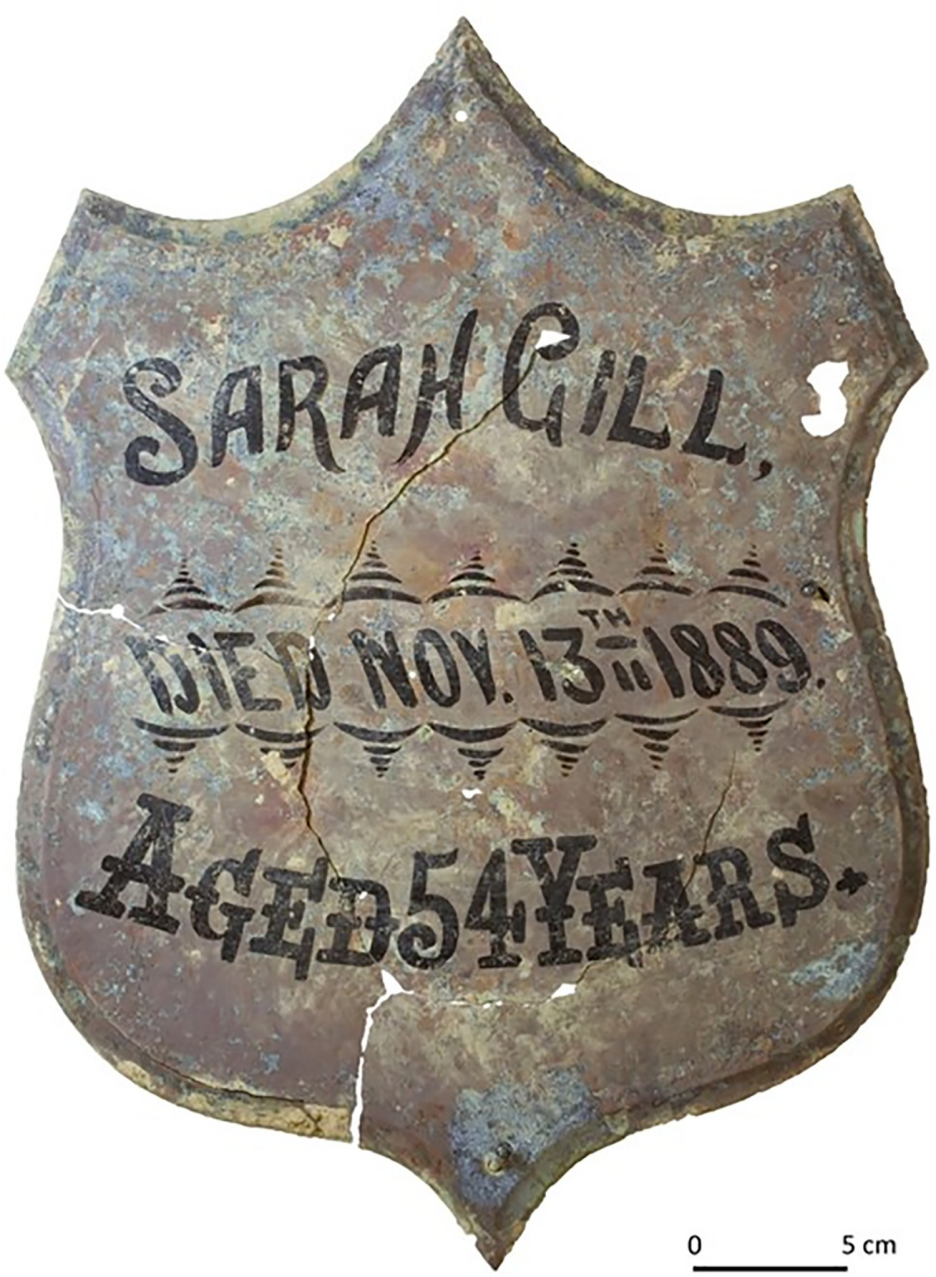
Example of a coffin plate excavated from the cemetery.

Fewston is a small village located in the Washburn Valley, near Harrogate in North Yorkshire, UK (Fig 1). In 2009–2010 a skeletal assemblage comprising 154 skeletons was excavated from the parish churchyard in advance of building work [22, 23]. The graveyard was in use from at least the 14^th^ century and closed in AD 1896. A relatively small area (approximately 300 m^2^) was excavated, and coffin plates/headstones indicated that the better-surviving burials in this part of the churchyard were predominantly of 19^th^ century date. This site was exceptional because very few rural churchyards of this date have been excavated (due to an urban bias in development) and 22 of the skeletons (21 adults and 1 infant) were of known identity due to associated coffin plates and/or grave monuments (Fig 2). The identified individuals were mostly born in the first half of the 19^th^ century. Birth and death certificates along with census records were obtained for each named person, revealing that most had spent their childhoods locally in the vicinity of the Washburn Valley, an area underlain predominantly by Carboniferous sandstones. This assemblage therefore provided the additional rare opportunity within an archaeological context to establish the local isotope signal from contemporaneous individuals known to have grown up in the area, on a lithology unfavourable to bone preservation and for which there are very few comparative cemetery populations.

**Fig 3:**
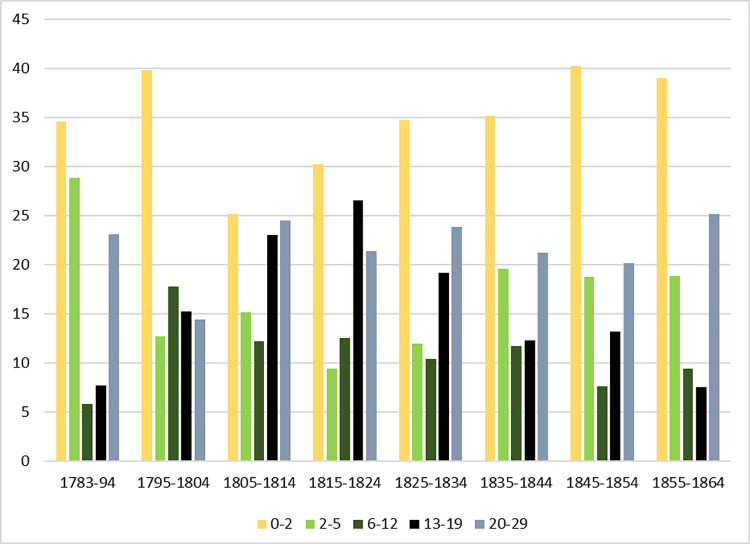
Burial records for Fewston church showing the dramatic increase in deaths amongst those aged 13–19 years (black bars) during the height of the mill’s productivity in the early 19^th^ century.

Analysis of the skeletal remains revealed that 51 individuals were less than 18 years of age [23], and that there was an unusually high proportion of juvenile and adolescent skeletons [24]. In the 18^th^ and 19^th^ centuries, there were five mills in the upper Washburn Valley involved in the processing of flax, cotton, or silk, with West House mill being one of the largest in the Yorkshire Dales [25]. Burial records from Fewston church show a peak in adolescent deaths during the mills’ most productive years (in the early part of the 19^th^ century). Between 1805 and 1824, the deaths of 29 children (aged less than 18 years) were recorded with the address of West House, indicating the mill (Fig 3). A similar observation was made by John Haynes Holmes [26], a visiting Unitarian Minister from the United States, who states:

“When examining the parish register, on the occasion of my visit to the Fewston church in the summer of 1913, I was struck by the large number of "deaths" recorded at ages from nine or ten to eighteen or twenty years. It seemed as though, in these early days in the dale, a wholly disproportionate number of persons died in their youth. The vicar, who was showing me his church and neighbourhood, suggested tuberculosis; I suggested child-labour” (p.48)

Historical documentation confirms that the mill employed significant numbers of pauper children, some of whom were recruited from workhouses in Hull and others from the impoverished London parishes of Southwark and Lambeth (Table 1, data from Honeyman [7]).

**Table 1:** Parishes known to have sent children to the West House Mill, Fewston (data taken from Honeyman [7] (p. 268).

Parish	Date	Girls	Boys
St George the Martyr, Southwark, London	1803	11	0
Lambeth, London	1803	4	0
St George the Martyr, Southwark, London	1804	14	6
St Leonards, London	1811	3	14
Lambeth, London	1811	3	0
Sculwater, Hull	1814	7	0
St George the Martyr, Southwark, London	1814	7	10

As Table 1 shows, both boys and girls were indentured to West House Mill. For example, in 1803 Caroline Farmer was one of the youngest apprentices at 7 years of age, sent from the parish of Southwark in London. In September of that same year, Sarah Canty, aged 12 years, was apprenticed to Fewston from Lambeth in London, along with three other girls of a similar age. In November of that same year Sarah’s brother Cornelius, who was only 7 years of age, was sent to a mill in Lancashire [27]. The West House mill owners, Colbeck and Company, built sex-segregated apprentice houses nearby specifically to accommodate the wholesale employment of pauper children that were so essential to the economic viability of the mill [7].

A first-hand account of child labour within the Fewston mill was written by the Reverend Robert Collyer [28], whose parents had both been pauper apprentices, originating from Norwich and London. Robert worked in the mill from the ages of 8 to 14 years and describes the conditions in his memoir:

“[we were]…rung in at 6 in the morning and out at 8 in the evening with an hour for dinner and a rest. And if we got a chance to sit down for a few moments when the overlooker wasn’t around to lay his leathern strap on our small shoulders….and the result of this was that weaker children were so crippled that the memory of their crooked limbs still casts a rather sinister light for me on the scriptures” (p.15-16).

Collyer proceeds to describe his constant tiredness (“tired beyond all telling”) and dread of being woken by the “infernal” bell ringing him to work [28]. This and other accounts of the lives of pauper apprentices such as *The Memoir of Robert Blincoe* [14] provide insights into the hardships that these children endured. There has, however, been no opportunity for independent bioarchaeological analysis of the impact of poverty and labour on their growing bodies. We hypothesise that the excess in juvenile and adolescent deaths within the cemetery sample was due to the influx of migrant child workers, as pauper apprentices, who subsequently died during their period of indenture. This multi-method analysis aims to verify that some of the non-adult skeletons from Fewston were pauper apprentices and to provide direct osteobiographical evidence of their lives and deaths.

## Materials and methods

### Osteological analysis

A total of 154 individuals from the Fewston cemetery were analysed. The Department of Archaeology, Durham University, Ethics Committee reviewed and provided written approval of the research design. The ethical guidelines of the British Association for Biological Anthropology and Osteoarchaeology [29] for the analysis of human skeletal remains were followed. Adult volunteer researchers participated in this project, including descendants of the named individuals excavated from the site. Volunteers and descendants provided verbal and written consent for the project activities. All skeletons were reburied following analysis, in a ceremony that included contributions from descendants, scientists and the local community.

For the purposes of this study ‘non-adults’ refer to those less than 20 years of age, rather than the more usual 18 years. This was to ensure the inclusion of all those who may have been pauper apprentices (indentures generally lasted until 21 years). Skeletal preservation was recorded by Caffell and Holst [23] using the McKinley [30] grading system. Overall, there was moderate to severe fragmentation of skeletons, with 40% of individuals severely fragmented, and one-third of skeletons less than 20% complete (only 16% were over 80% complete). Age at-death for the non-adults was estimated using dental development [31–33]. Other skeletal indicators of age were recorded, including long bone growth [34–36] and epiphyseal union [36]. Due to the disparity between the dentition and post-cranial indicators of age within this assemblage, the dental evidence was prioritised for age estimates as this is known to correlate most closely with chronological age. Sex of the non-adults was not estimated using morphological or metrical techniques prior to puberty (e.g. Schutkowski [37]), as these are considered unreliable [38]; instead, sex estimation was undertaken for 11 non-adults using the amelogenin peptide method [39] (see below).

For those aged approximately 18 years and over, sex was determined using the standard morphological analysis of the pelvis and skull [40–42]. Age at death of adults was estimated from observations of late-fusing epiphyses [36], morphological changes of the pubic symphyseal face [43], auricular surface [44] and ribs [45–47]. In this study, the category of ‘adult’ was used for those over the age of 20 years, but for whom no specific age category could be assigned due to poor preservation.

Pathological analysis was undertaken to record metabolic disease, including vitamin C and vitamin D deficiencies [48–51] evidence of upper and lower respiratory tract inflammation in the form of woven and lamellar bone in the maxillary sinuses and on the visceral surfaces of the ribs, and signs of infectious disease [50–52]. Dental enamel defects were recorded in both the deciduous and permanent dentition [53–55].

### Amelogenin peptide analysis

Several studies in recent years have successfully demonstrated that proteins can be extracted from human tooth enamel and used as a method for identifying the sex of archaeological individuals [39, 56–60]. Tooth enamel is formed by a small number of proteins which then become trapped in the mineral as it matures. These residual proteins are primarily comprised of the heterogeneous, dimorphic amelogenins (AMELX and AMELY), which can be extracted and identified from both deciduous and permanent teeth. For this study, teeth from eight non-adults were analysed using the method described by Stewart and colleagues [39], which uses a minimally destructive acid etching procedure and nano liquid chromatography tandem mass spectrometry (see S1 Appendix for full details of the procedure). Amelogenin peptide results for three Fewston non-adults analysed previously using the same method have also been included here [61].

### Isotope analysis

A single tooth from 31 individuals, including 11 named adults, were sampled from Fewston for ^87^Sr/^86^Sr and δ^18^O. Strontium isotope migration studies are based on the principle that specific types of bedrock produce strontium isotope ratios that are characteristic and indicative of the underlying geology, and these are transmitted through soils to the food chain [62]. However, a simple relationship between biological samples and underlying bedrock does not exist due to factors such as differential weathering of minerals, drift deposits, dust deposition and rainwater inputs, any of which can shift the isotope ratios away from whole-bedrock compositions [63, 64]. Humans may also source food from multiple locations. However, as food and water in the past are likely to have been primarily locally sourced, human strontium isotope ratios that differ from locally obtained values of other humans, animals or plants are presumed to indicate migrants [62].

Oxygen incorporated in the human body is derived from breathed oxygen and approximately 80% is from ingested fluids, from drinking water and food, which in past populations mostly derived from local precipitation. The δ^18^O of human bioapatite is directly correlated with ingested water [65]. Whilst atmospheric oxygen has no geographical variation, the δ^18^O of precipitation decreases as latitude, distance from the coast, or altitude increase [66, 67]. Northwest Europe’s climate and wind system create a specific southwest-northeast pattern across Great Britain [66]. Oxygen isotopes incorporated into body-water are fractionated from their sources in breathed oxygen and ingested water, and fractionated again as they are incorporated into tissues such as tooth enamel. Consequently, calibration equations are required to infer drinking water, and thus local precipitation, values from measurements on tooth enamel carbonate (O_c_) or phosphate (O_p_) [68–70].

Strontium and oxygen are incorporated into enamel during mineralisation, thus the isotope ratios reflect childhood residence [71]. Wherever possible enamel from second premolars (P2) or second molars (M2) was sampled, selecting teeth with at least one surface of the tooth unaffected by dental caries, and no extreme wear or damage. Enamel hypoplasia, often present in the adolescent dentitions in this sample, was an exception to these selection criteria. A small chip of enamel was mechanically cleaned by abrading all surfaces by a minimum depth of 100μm with a tungsten carbide dental burr and was then hand-ground with an agate mortar and pestle to produce two samples of core enamel between 10 and 20 milligrams in weight [71, 72]. Measurements were undertaken at the Department of Earth Sciences, Durham University.

Strontium was extracted according to published methods [73] and measurements made using a Thermo Neptune plasma ionisation multicollector mass-spectrometer. The standard NBS987, gave a value of 0.710265 ± 0.000007 (n = 11, 2σ), producing an external reproducibility of ± 0.0024% during the analysis of these samples. All strontium isotope ratios were corrected to a NBS987 standard value of 0.710250 [69].

Oxygen isotope analysis of enamel carbonate (O_C)_ followed the methods of Bentley and colleagues [74]. Approximately 2 mg of powdered enamel were analysed using a Thermo Fisher Scientific Gasbench II with a Thermo Fisher Scientific MAT 253 gas source mass spectrometer. All samples were analysed in duplicate yielding a technical error of measurement of 0.19 ‰ for δ^18^O. International (NBS 18, IAEA-CO-1, LSVEC) and in-house laboratory (DOBINS, DCS01) standards indicated that analytical reproducibility was ±0.11 ‰ (1 s.d.) or better for δ^18^O. For comparisons with drinking water and other published data, δ^18^O_C_ values were converted to δ^18^O_p_ using equations from Chenery and colleagues [70].

Carbon (δ^13^C) and nitrogen (δ^15^N) stable isotopes of bone collagen provide information on the main protein sources in the diet [75], averaged over the time of bone formation and turnover, which may represent well over a decade in adults, depending on skeletal element [76]. Carbon isotope values distinguish the consumption of C_3_ plants, which include most crops in a temperate environment including wheat, oats and barley, from C_4_ plants, such as millet and maize [77]. Maize is recorded as an import during the 19^th^ century as either a poor or famine relief food in Ireland [78, 79], or for use as animal fodder [80 (p322)]. Nitrogen isotope values indicate the trophic position of an individual, with a stepwise enrichment of ^15^N occurring along the trophic chain from plants to herbivores to carnivores, usually resulting in δ^15^N values increasing by 3–5‰ [81]. Carbon isotope values also increase by trophic level but to a lesser extent, typically 0–2‰. Carbon and nitrogen isotope values can be used together to identify the consumption of terrestrial or aquatic resources, where marine resources in particular exhibit enrichment in both ^13^C and ^15^N [82].

Rib samples from a total of 74 human individuals representing a range of age categories (0–1 year, 1–7 years, 8–20 years, +20 years), including the identified/named adults (+20 years) were sampled from Fewston for δ^13^C and δ^15^N. Seventeen animal bones comprising cow, sheep, pig, chicken and dog were also sampled from post-medieval (16^th^-19^th^ century) contexts from the site of Hungate, York to provide a comparative animal baseline. The carbon and nitrogen isotope analysis was carried out at the facilities at BioArCh at the University of York. Collagen extraction from bone followed a modified Longin method [83] with an additional ultrafiltration step [84] following the method described in Bleasdale and colleagues [85]. Collagen was analysed in duplicate using a Sercon 20–22 continuous flow isotope ratio mass spectrometer coupled to a Sercon GSL elemental analyser. Accuracy was determined by measurements of international standard reference materials within each analytical run. These were IAEA (600 ẟ^13^Craw = -27.79 ± 0.1 ‰, ẟ^13^Ctrue = -27.77 ± 0.043 ‰, ẟ^15^Nraw = 0.82 ±0.19 ‰, ẟ^15^Ntrue = 1 ±0.2 ‰); IAEA N2 (ẟ^15^Nraw = 20.53 ± 0.16 ‰, ẟ^15^Ntrue = 20.3 ±0.2 ‰); IA Cane (ẟ^13^Craw = -11.8 ± 0.09 ‰; ẟ^13^Ctrue = -11.64 ±0.03 ‰). The overall uncertainties on the measurements of each sample were calculated using the Kragten method [86], combining uncertainties in the values of the international reference materials and those determined from repeated measurements of samples and reference materials, expressed as one standard deviation. The maximum uncertainty for all samples across all runs was <0.3 ‰ for both ẟ^13^C and ẟ^15^N. A homogenised bovine bone was also extracted and analysed within the same batches as the samples and produced the following average values; ẟ^13^C = -23.0 ± 0.2 ‰; ẟ^15^N = 6.6 ± 0.3 ‰. This was within the overall mean value from 50 separate extracts of this bone sample, which produced values of ẟ^13^C = -23.1 ± 0.3 ‰ and ẟ^15^N = 6.5 ± 0.5 ‰.

Statistical analysis of ẟ^13^C and ẟ^15^N data was conducted using a Mann Whitney U test for pairwise comparison between sexes. For comparisons between multiple age categories, a Kruskall Wallis test was used with additional Dunn’s post-hoc tests and Bonforri corrected p values to adjust for multiple testing. Statistics were carried out using PAST version 4.07 [87] and RStudio Version 1.4.1717.

## Results

### Demographic data

**Fig 4:**
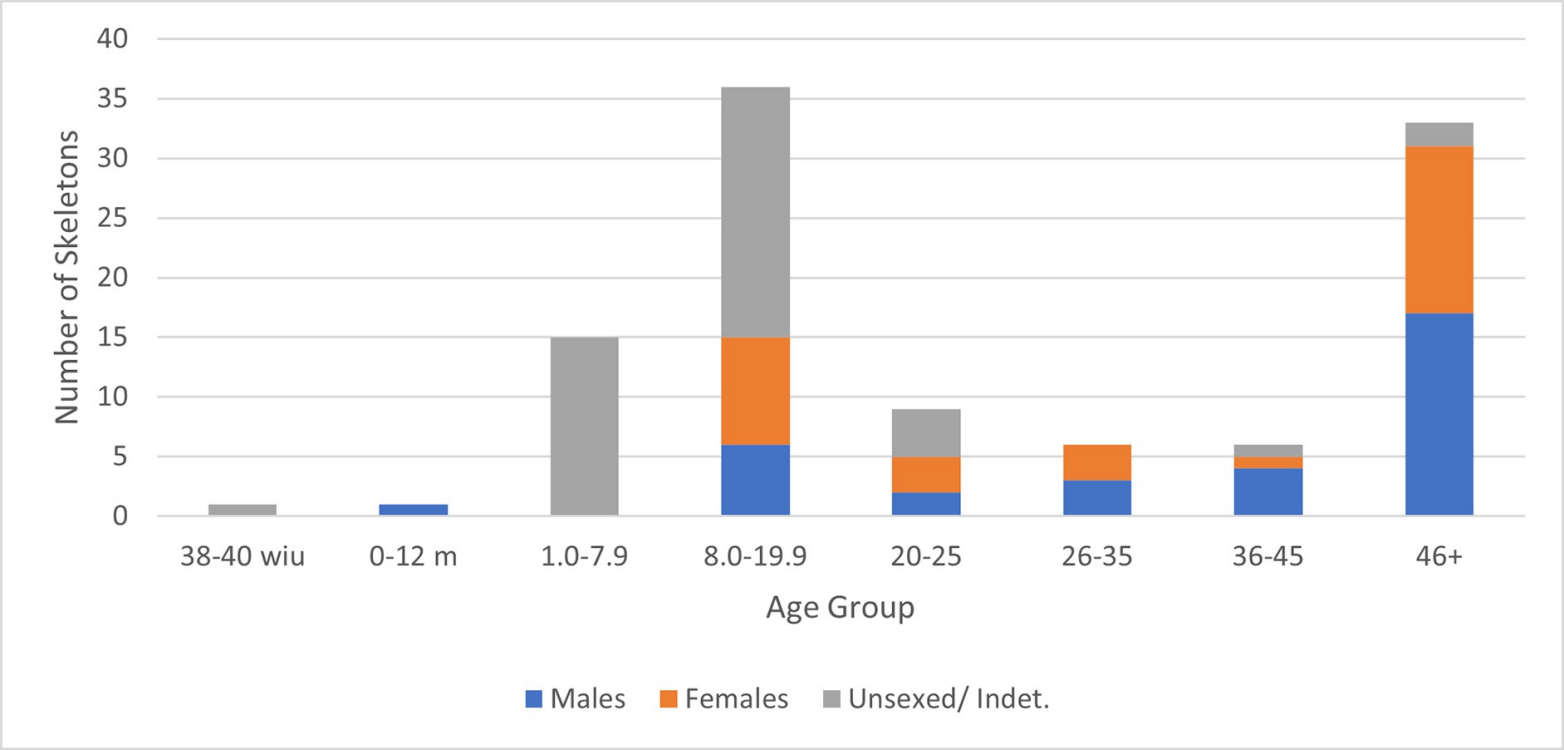
Age and sex distribution of the Fewston skeletal sample. The sexes of the non-adults include the 11 peptide skeletons, 1 coffin plate skeleton, and four older teenagers who were sexed morphologically.

The demographic profile of the Fewston site highlights the unusually high proportion of individuals aged 8–20 years–the ages of indenture for pauper apprentices (Fig 4). This is an atypical demographic profile: most cemetery sites show greater mortality in children less than 6 years of age, and very few adolescents, reflecting the greater vulnerability of younger children to infectious diseases. Overall, there are equal numbers of males and females, but of those aged 8–20 years for whom sex was estimated, there is a bias towards females (60:40). The demographic data is consistent with the historical data for Fewston, showing both an elevated number of individuals between 8–20 years and a similar ratio of girls to boys in terms of those recruited to the Fewston mill (ratio of girls to boys was 62:38).

### Strontium and oxygen isotope data

**Fig 5:**
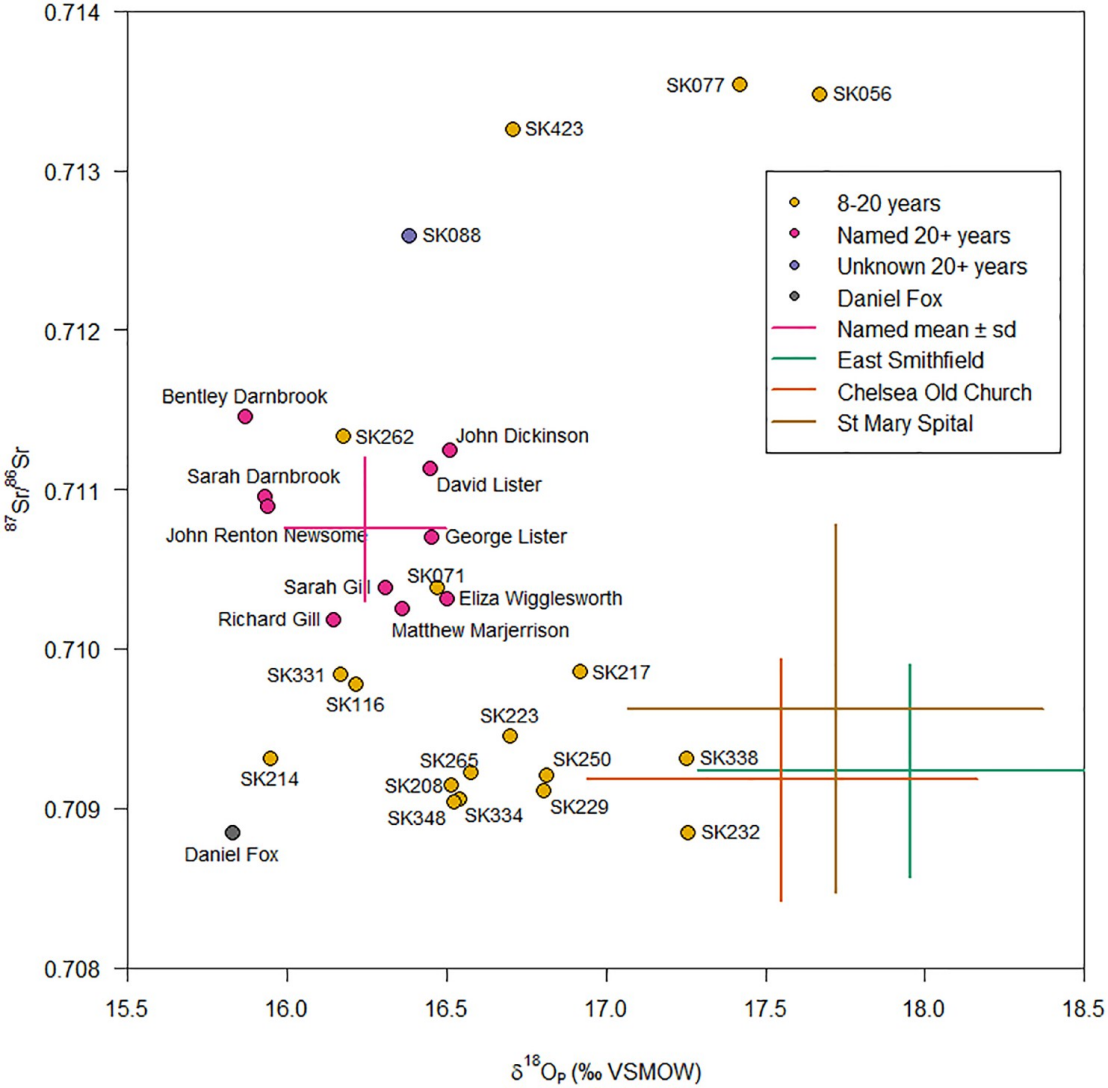
Strontium and oxygen isotope profiles of the named adults and those aged 8–20 years. Daniel Fox is labelled in an open orange square because prior to analysis his identity was not completely certain.

Fig 5 and Table 2 show the strontium and oxygen isotope ratios for the named individuals and a sample of the unknown non-adults aged between 8–20 years from Fewston (full details are provided in S1 Appendix). These values will relate to the pre-indenture period of the children’s lives, as the crowns of the permanent second premolars and second molars are completed by approximately 6.5 years and 8.5 years, respectively [33]. The data for the named individuals provides an unusually well-provenanced mean and range for both strontium (mean = 0.7107, range 0.7102–0.7115) and oxygen (mean = 16.2‰, range 15.9–16.5‰) isotopes of people originating from a region of Carboniferous sandstone in the north of England for which there is currently few comparative archaeological datasets. Interestingly, and highly unusually for most archaeological cemetery populations where young children tend to define the local range most reliably, most of those aged 8–20 years demonstrate isotopic characteristics distinct from the identified local individuals, who cluster relatively well together. Whilst we cannot be sure of the precise origins of these 8-20-year individuals, those showing strontium isotope ratios below 0.710 are consistent with values from London, as illustrated by comparisons with the mean values from three populations in London: St Mary Spital, East Smithfield [88] and Chelsea Old Church [89]. The isotope data therefore correlates well with the historical records, which suggest that many of the West House mill apprentices had come from the parishes of Lambeth and Southwark in London (Table 1).

**Table 2:** Summary statistics for the strontium and oxygen isotope values categorized by ‘named’, ‘unknown adult’ and ‘adolescent’ individuals from Fewston.

		δ ^87^Sr/^86^Sr (‰)				δ ^18^O_p_ (‰)			
Age/Name d status	No.	Mean	1 σ	Min	Max	Mean	1 σ	Min	Max
8.0–20 year-olds	19*	0.71013	0.00158	0.70885	0.70986	16.7	0.46	15.9	17.7
Named Adults	11	0.71058	0.00072	0.70885	0.71146	16.2	0.27	15.8	16.5
Unknown Adult	1	0.71259	-	-	-	16.4	-	-	-

*8-20-year-old group has 16 individuals for Sr and only 15 individuals for oxygen.

Prior to analysis one individual had only been tentatively identified, so was designated as an ‘unknown adult’. The tentative identification was as ‘Daniel Fox’, with historical records revealing that he was originally from Dungannon, Co. Tyrone in Ireland, and then moved to North Yorkshire, joining the West Riding Constabulary in 1872. Daniel died in 1884 aged 33 years [90]. The low isotope ratio supports this skeleton’s identity as Daniel Fox, being inconsistent with the Carboniferous sandstones at Fewston but consistent with the range predicted around Dungannon [91] and the Carboniferous limestone rocks on which it is located [92].

There are also three 8-20-year-olds with strontium isotope ratios over 0.713. Biosphere ratios this high have predominantly been reported or predicted from regions underlain by Palaeozoic, and older, silicate rocks which are found in the west and north of Britain such as the Malvern Hills [69], the Old Red Sandstone regions of Herefordshire, parts of the Lake District and the Scottish Highlands [93], areas of south Wales [94], the West Country [95], southeast Ireland and scattered areas in the north of Ireland [91]. There are also groups of prehistoric people with similarly high ratios in the Peak District and Yorkshire which cannot be explained by the underlying geology [96]. At present, further research is required to clarify the possible origins of these individuals.

An unnamed adult male, SK 088, also exhibited distinctive isotope values when compared to the named individuals. This individual had suffered a sharp force injury to his cranium, which was healed, as well as numerous healed fractures of his ribs, vertebrae and sternum; he had also been buried wearing boots with distinctive pearl buttons (date c. 1825) and with a George III coin (date 1818). Archival research suggests that his identity might be a 31-year-old soldier named Timothy Callaghan, buried in 1849, and described in the burial record as ‘a stranger’ whose body was found in a rural outhouse. Timothy’s cause of death was recorded as a ‘visitation by God’, suggesting a quick unexplained death [97]. It is interesting, therefore, that his isotopic signature also distinguishes him as ‘other’, as well as his clothing and palaeopathological evidence.

### Carbon and nitrogen isotope data

**Fig 6:**
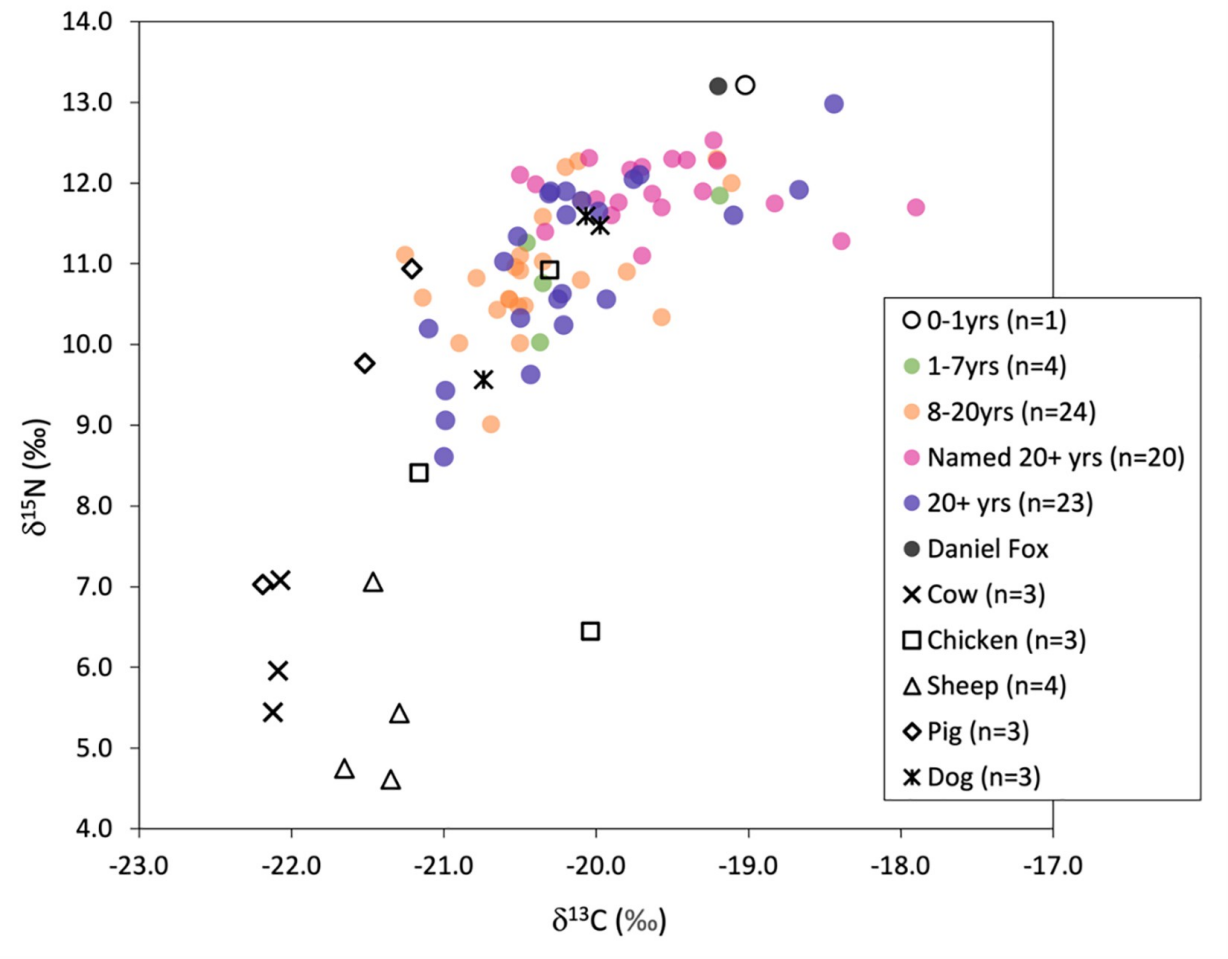
Scatterplot of humans from Fewston with animals from Hungate, York with the outlying pig specimen with a δ^13^C value of -10.2‰ omitted to allow for greater clarity in the plot.

**Fig 7:**
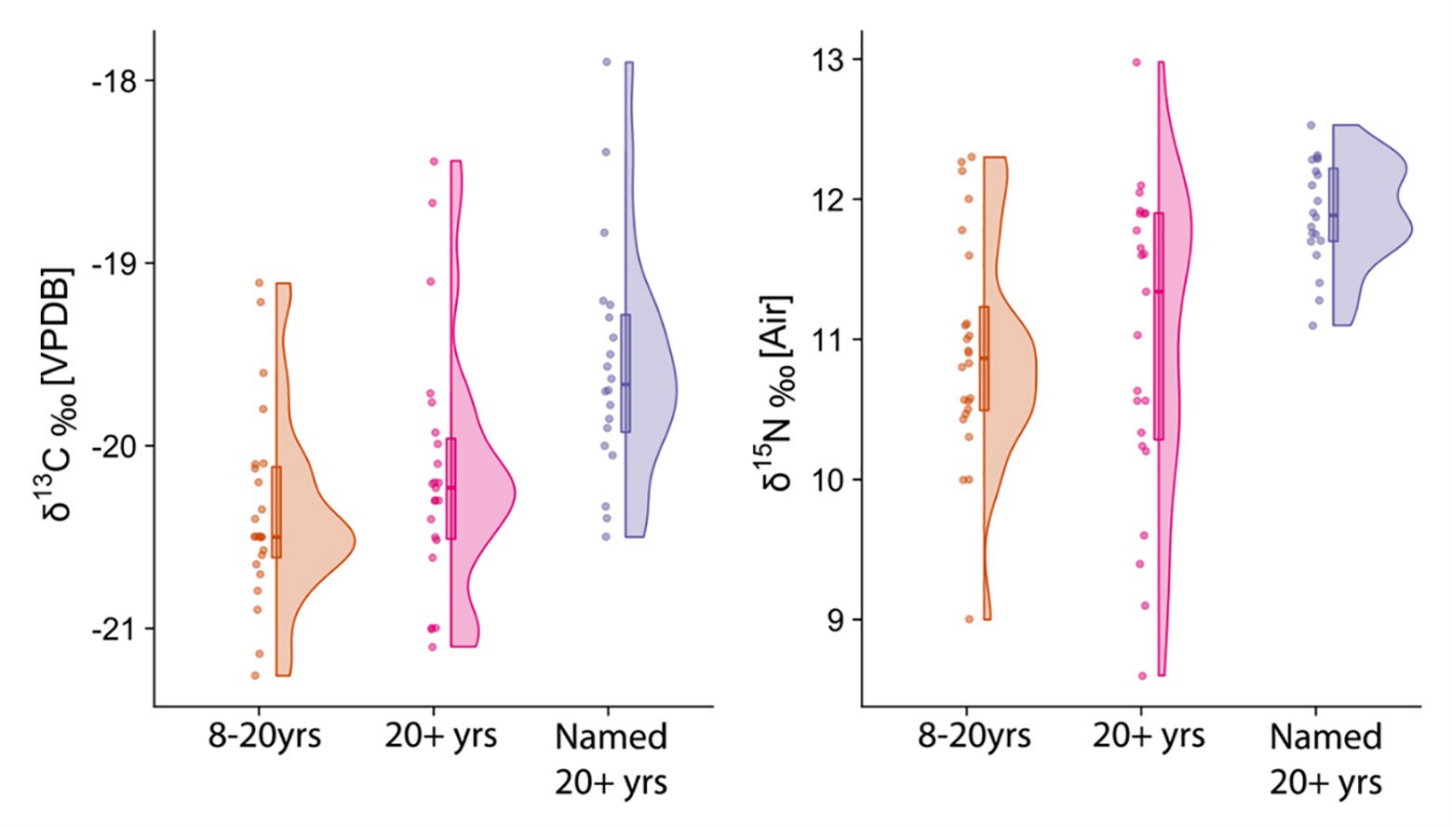
Raincloud (boxplot + violin + jitter) plots [99] of human data categorized by age or named status for group sizes >5.

The full results for δ^13^C and δ^15^N isotope values for humans and animals, including collagen quality indicators (C:N, %C, %N) can be found in the supplementary information (S2 Appendix). The carbon and nitrogen isotope data for the sampled individuals are plotted in Figs 6 and 7, separated by age category and named status. The summary statistics for each group is presented in Table 3 and for different animal species from Hungate York, as a comparative baseline, in Table 4. The vast majority of individuals aged between 8–20 years and three of the four individuals aged between 1–7 years notably plot together in the lower end of the range of δ^13^C and δ^15^N values, indicating that their diet for the 3–5 years preceding death was lower in animal protein compared to the majority of the adult population. The named adults immediately stand out for possessing a tighter range of higher δ^13^C and δ^15^N values, indicative of diets with a larger proportion of high trophic level animal protein when compared to unnamed adults and the individuals younger than 20 years of age. The unknown adults (20+ years), by contrast, possess a wide range in isotopic values (δ^13^C = 2.7‰, δ^15^N = 4.4‰), encompassing the range of isotopic values possessed by both the named adults and non-adults. This likely reflects the presence of individuals of different social status and potentially the presence of non-locals amongst this group. The offset in δ^13^C and δ^15^N between the humans from Fewston and herbivores from York reflect the differentiation in animal protein consumption (the different geographical locations notwithstanding), with the named adults possessing the highest values of >2‰ for Δ^13^C_human-herbivore_, and 5‰ for Δ^15^N_human-herbivore_ (Table 3), just above the typical trophic offset of 3–5‰ [81] and therefore indicating a more significant input of protein from omnivorous animals such as chicken, pigs or potentially some minor input of freshwater or marine sources (Table 4).

**Table 3:** Summary statistics for humans from Fewston by age category and named status.

		δ^13^C (‰)					δ^15^N (‰)				
Age (yrs)/Named status	No.	Mean	1 σ	Min	Max	Human-herbivore offset*	Mean	1 σ	Min	Max	Human-herbivore offset*
0–1	1	-19.0	-	-	-	-	13.2	-	-	-	-
1–7	4	-20.1	0.6	-20.5	-19.2	1.6	11.0	0.8	10.0	11.9	5.2
8–20	24	-20.4	0.5	-21.3	-19.1	1.3	10.9	0.8	9.0	12.3	5.1
Named 20+	20	-19.6	0.6	-20.5	-17.9	2.1	11.9	0.4	11.1	12.5	6.1
Unnamed 20+	23	-20.2	0.7	-21.1	-18.4	1.5	11.0	1.1	8.6	13.0	5.2

*Human-herbivore offset (Δ^13^C, Δ^15^N) calculated using the mean δ^13^C and δ^15^N for sheep and cattle combined: -21.7 and 5.8‰ respectively, n = 7). An offset was not calculated for the infant individual Rowland Marjerrison who may have been breastfeeding at the time of his death at 10 months. The Named 20+ category only includes locals, omitting named individual Daniel Fox, the only named non-local.

Statistical analysis indicates the isotopic differences between age categories and the category of ‘named’ individuals, where group size is >5 are significant for both δ^13^C and δ^15^N (H (2):17.66 p = 0.00, and H (2): 16.5, p = 0.00 respectively) and post-hoc comparison indicates that, as anticipated, named adults stand significantly apart from individuals in the 8–20 year and unnamed adult category (S2 Appendix). There was no statistically significant difference in isotopic values between males and females for either δ^13^C or δ^15^N within each age category (8-20yrs, named 20+ years and unnamed 20+ years, S2 Appendix).

**Table 4:** Summary statistics for animals from Hungate, York, by species used as a comparative baseline.

		δ^13^C (‰)				δ^15^N (‰)			
Species	No.	Mean	1 σ	Min	Max	Mean	1 σ	Min	Max
Cattle	3	-22.1	0.0	-22.1	-22.1	6.2	0.8	5.4	7.1
Sheep	4	-21.4	0.2	-21.7	-21.3	5.5	1.1	4.6	7.1
Pig	4	-18.8	5.7	-22.2	-10.2	8.9	1.8	7.3	10.9
Dog	3	-20.3	0.4	-20.7	-20.0	10.9	1.1	9.6	11.6
Chicken	3	-20.5	0.6	-21.2	-20.0	8.6	2.2	6.4	10.9

A few of the named adults possess δ^13^C isotope values approaching -18‰ including SK360 (Gill Wigglesworth) who has δ^13^C and δ^15^N isotope values of -17.9‰ and 11.7‰ respectively, indicative of the consumption of C_4_ crops directly or, perhaps more likely given the corresponding δ^15^N isotope values, C_4_-fed animal protein. Low trophic level marine foods are unlikely given the rarity of marine fish in Fewston [98], although it cannot be ruled out and the possibility of dried/smoked fish consumption must be considered. One pig from York, however, had a remarkably high δ^13^C isotope value of -10.2‰ (not shown on the graph, see S2 Appendix), confirming that animals fed on C_4_ crops such as maize was a potential source of ^13^C enrichment in humans.

**Fig 8:**
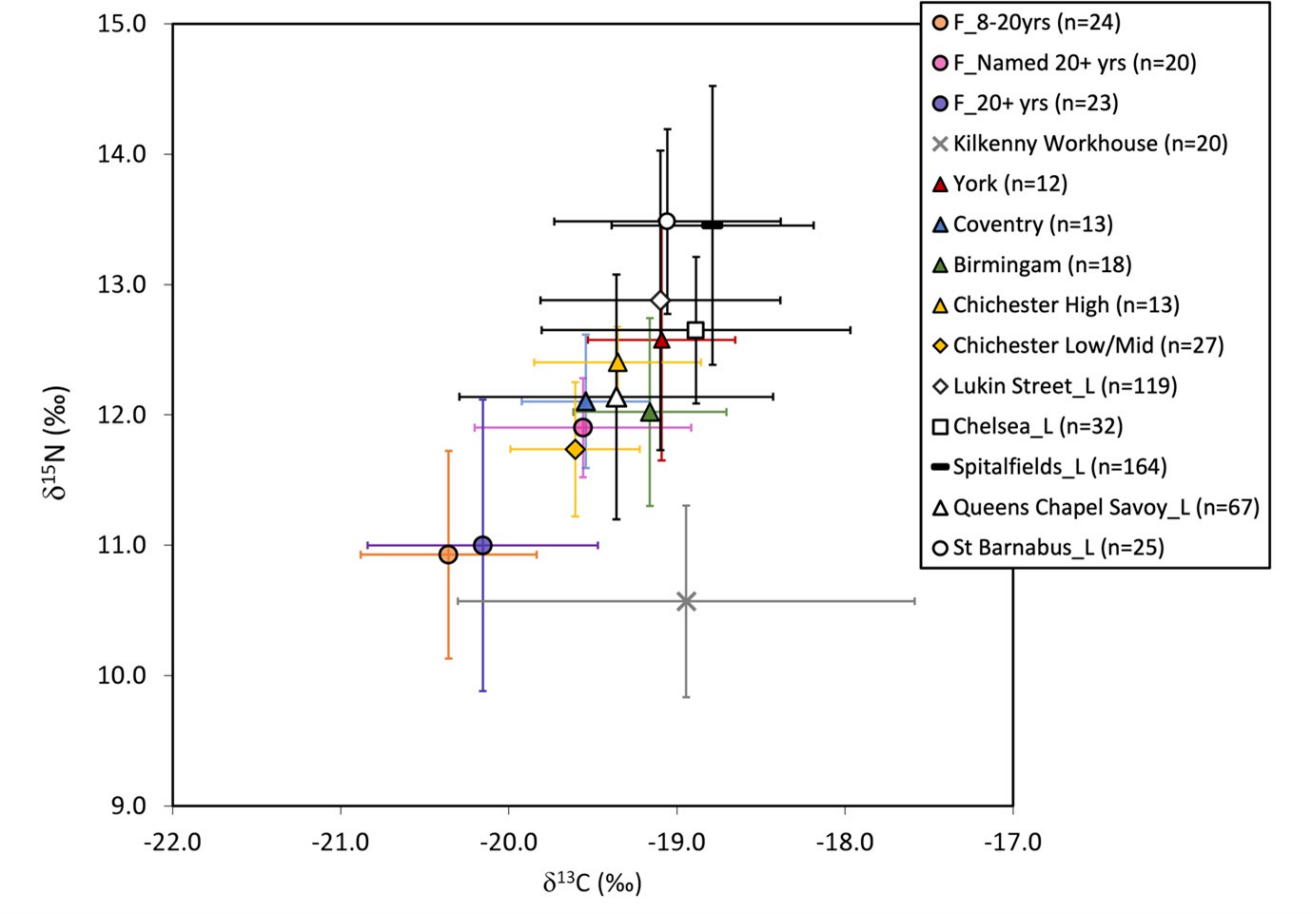
Plot of mean and standard deviations for δ^13^C and δ^15^N in populations previously studied from the post-medieval period against age categories from Fewston where sample size >5 individuals (denoted with prefix F). Sites in London are denoted by ‘_L’. Reference populations derive from: York [100], Coventry and Chelsea [89], Birmingham [101], Lukin Street and Kilkenny Workhouse [78], Chichester low/mid status and high status denoted by burial type [102] (S2 Appendix), Spitalfields [103], Queen’s Chapel Savoy and St Barnabas [85]. Comparative data can be found in S2 Appendix.

The average isotopic values for the individuals aged between 8–20 years and unnamed adults (20+ years) from Fewston are not just low in comparison to named adults at Fewston, they are also distinctively low compared to other published post-medieval populations (Fig 8). Their δ^15^N isotope values are not far from those from Kilkenny workhouse, Ireland who represented the Irish rural poor and consumed restricted, almost vegetarian diets [78]. This is unlikely to be solely an artifact of baselines, given that the named adults local to Fewston have significantly higher δ^15^N isotope values, although it cannot be ruled out entirely as animal remains from Fewston are lacking. The animal remains from Hungate in York, however, have isotopic values comparable to those published from London: the mean δ^15^N values for sheep/goat are slightly higher in London but by less than 1‰ [85]. The published isotope data for this period derive from predominantly urban populations of varying wealth and status. The named individuals from Fewston, a rural area, possess isotopic values similar to those of low to middling status at Chichester and middle-class populations in Birmingham and Coventry and also the population from Queen’s Chapel Savoy in London, but this cemetery represents an unusual mix of social status groups, including the military, a hospital and a prison [85]. Even the poor within these populations from towns and cities, particularly London as a major port, would have had access to a greater range of foodstuffs, including fish, afforded by the development of water and rail transport links. It is therefore perhaps not surprising that the pauper apprentices from Fewston, by comparison, stand apart as their isotope values will represent at least some period of life within one such city.

### Palaeopathological data

In the original assessment of the skeletal data the children of Fewston immediately appeared anomalous in terms of their delayed growth and the frequency and severity of pathological lesions [24]. When integrated with the isotopic analysis, it is clear that the growth delays and severe pathologies are more commonly present in the 8-20-year-old individuals identified isotopically as non-local (Table 5). The prevalence of vitamin deficiency diseases such as rickets and scurvy were unusually high amongst the non-adults at Fewston at 45% and 38% respectively [24]. Of those children identified as ‘non-local’ based on their isotope ratios, 50% (6 of 12 individuals) exhibited evidence of rickets. In contrast, only two of the 21 named local individuals (9.5%) had evidence for vitamin D deficiency, both of whom were from the same family: Jane Marjerrison had possible residual rickets and her 10-month-old grandson Rowland Marjerrison had evidence for both rickets and scurvy. Vitamin deficiencies are more easily diagnosed in children and this factor is likely to account for some of the disparity between the named individuals and the non-local children, but the difference here is very marked and further explanation is necessary. The prevalence of vitamin D deficiency amongst the non-local children is consistent with observations amongst the urban poor from London during this period [54, 104, 105].

**Table 5:** Crude prevalence of rickets, scurvy and cribra orbitalia.

	Rickets		Scurvy		Cribra Orbitalia	
	n	%	n	%	n	%
Non-local ‘adolescents’ (excluding 071 & 262)	6/12	50.0%	6/15	40.0%	8/12	66.7%
8–20 years (overall)	8/22	36.4%	11/28	39.3%	15/23	65.2%
Named individuals	2/21	9.5%	1/21	4.8%	8/19	42.1%

The named individuals exclude Daniel Fox who was non-local.

**Fig 9:**
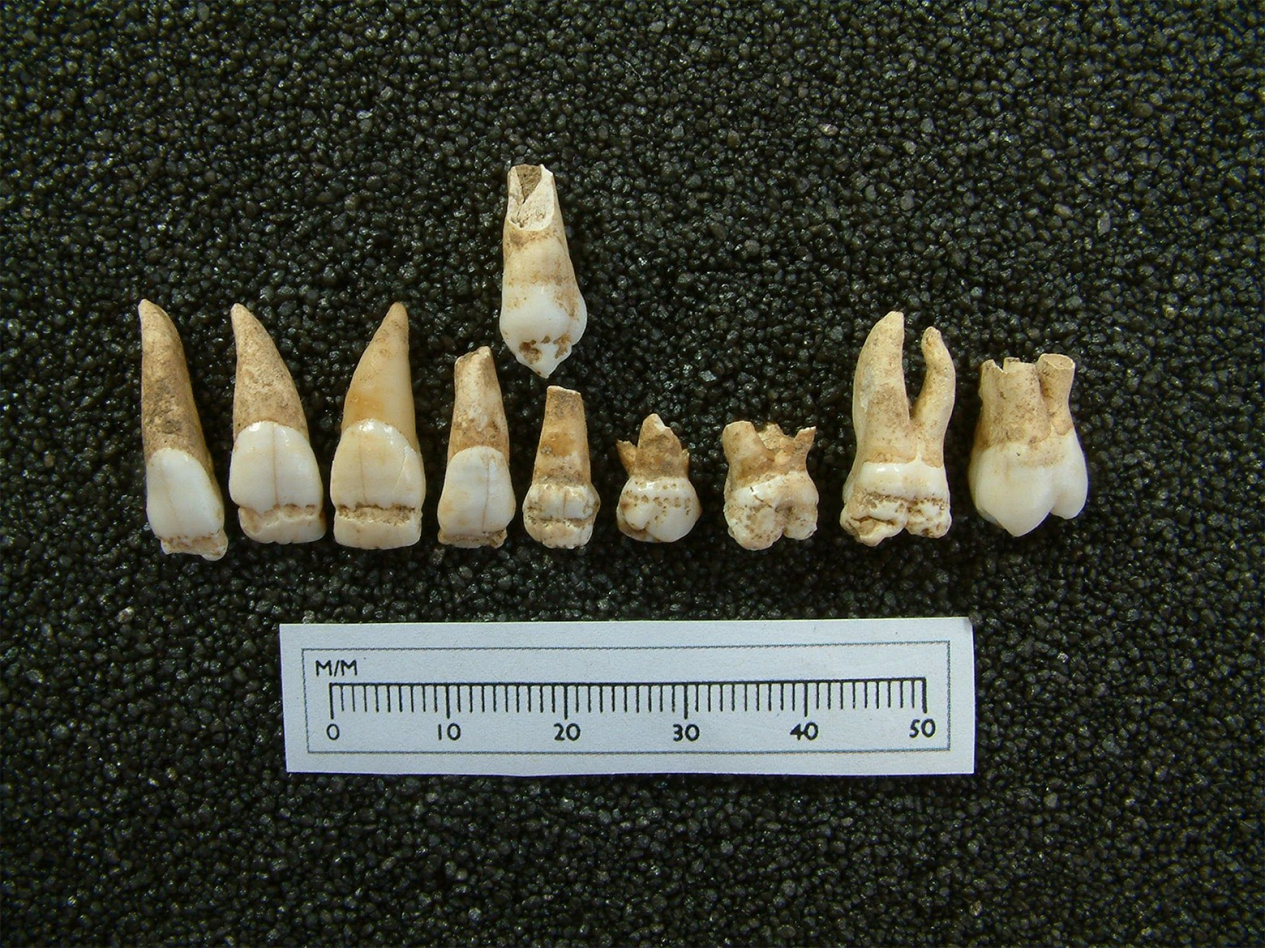
Dental enamel hypoplasia on the upper deciduous and permanent teeth of SK 232.

The deciduous and permanent teeth of 76% (13 of 17) of the isotopically ‘non-local’ 8–20-year-olds showed evidence of dental enamel defects. The crude prevalence rate of dental enamel hypoplasia amongst the named adults was, however, only slightly lower at 69%. Nevertheless, the nature of the defects observed in the non-adults was notably different, with 19% of non-local children showing evidence of severe plane form defects and/or cuspal enamel hypoplasia (Table 6, Fig 9). Enamel defects of this nature were not present amongst the named adult skeletons.

**Table 6:** Crude prevalence rates of isotopically identified non-local children aged 8–20 years (excluding Sk 071 and Sk 262 who may be local) with different forms of DEH.

Lesion Type	Deciduous			Permanent			Deciduous & Permanent		
	n	Total	%	n	Total	%	n	Total	%
Lines	0	5	0.0%	12	16	75.0%	12	16	75.0%
Pits	3	5	60.0%	7	16	43.8%	8	16	50.0%
Grooves	1	5	20.0%	3	16	18.8%	3	16	18.8%
Plane Form	1	5	20.0%	3	16	18.8%	3	16	18.8%

**Fig 10:**
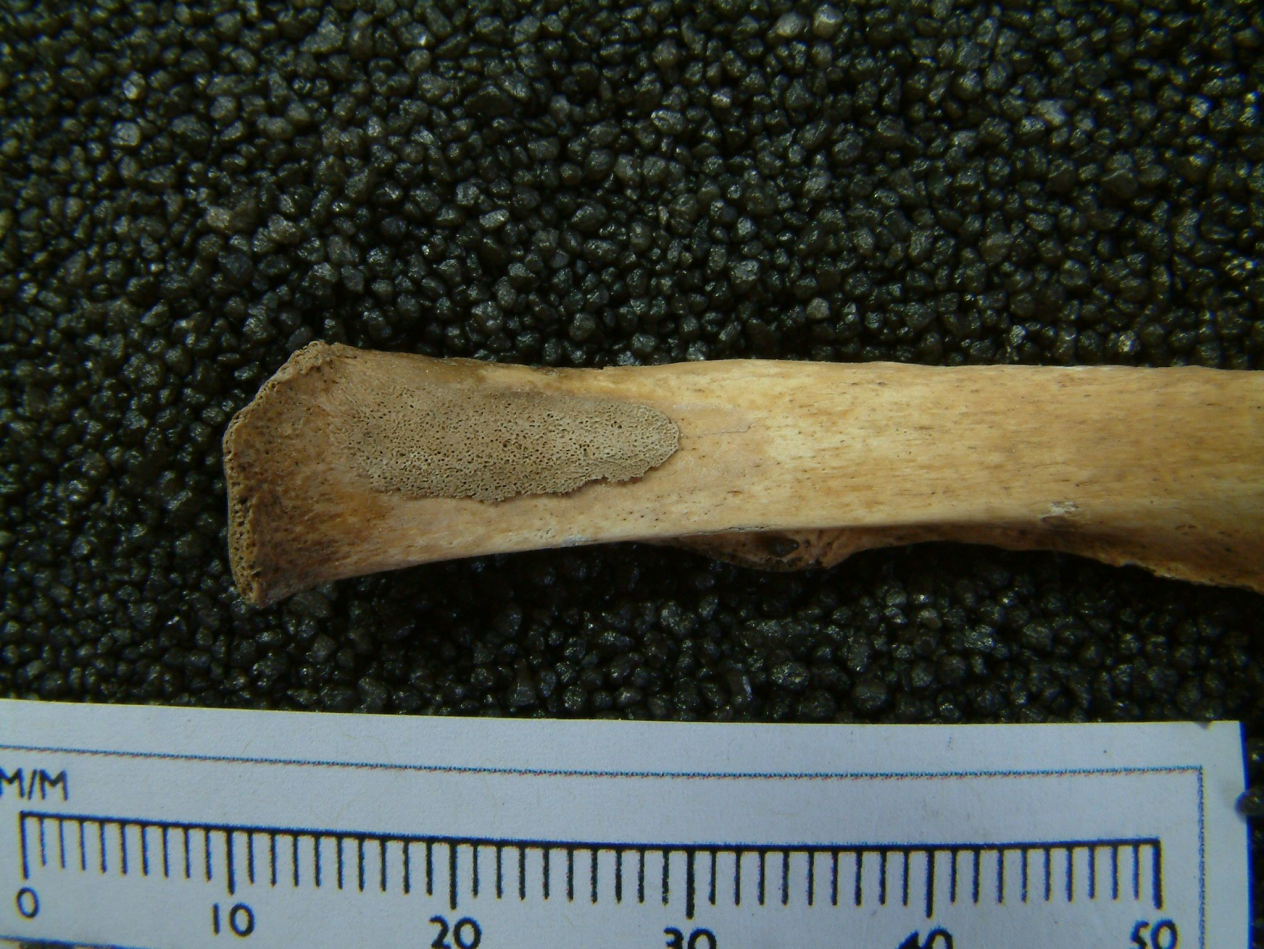
Woven bone on the visceral surface of the vertebral end of a left rib of SK 334.

A large number of the ‘non-local’ children showed evidence of new bone formation in the maxillary sinuses and/ or ribs: 57% exhibited evidence of maxillary sinusitis and 42% exhibited periosteal reactions to the visceral surfaces of the ribs (Fig 10). These are very high values, even when compared to urban sites of a similar period. One of the non-local children (skeleton 223, aged, 14–15 years) also had lesions in the cranium indicative of tuberculosis, a disease often associated with the urban poor and mill-workers. Non-local children also showed delayed long bone growth and epiphyseal fusion [24]. For example, SK 331 was a male aged 12–14 years based on dental development, but his long bone length was comparable with a child aged 7–8 years old. Skeleton 262, a probable female, was dentally aged 16–18 years, based on 3^rd^ molar development, but the long bones (still unfused) were of a size consistent with a child aged around 10 years; this individual also had 12 teeth with plane form dental enamel hypoplasia. SK 271 (sex unknown) was aged 17–19 years, but their long bone size was comparable with a child aged 9–13 years. In addition to growth deficits in the long bones, Gowland and colleagues [24] reported poor growth in vertebral body height for age amongst four of the Fewston adolescents with preserved vertebrae (SK208 (F), SK262 (F), SK331 (M), and SK338 (M)) when compared to a contemporary non-adult skeletal sample from northern England.

On average, the named adult males were 176.8 cm tall, and the named adult females were 159.7 cm tall (stature estimated from long bone length). The male average for named adults at Fewston falls well above the range of mean statures (168–174 cm) reported for post-medieval sites, while the female average is comparable with the range of means reported (156–164 cm [106]). In contrast, three of the non-local skeletons for whom epiphyseal fusion was complete (indicating the cessation of growth) and stature could be calculated were shorter than most of the local adults: two females were 148.2 cm and 152.6 cm tall, whilst a male was 169.6 cm tall.

This picture of ill health amongst the non-local children aged 8–20 years of age contrasts with the skeletal evidence from the local named adults, and the population sample overall. The 8-20-year-old individuals from Fewston were distinctive from the named adult sample in terms of their isotopic evidence for mobility and diet, as well with respect to their delayed growth and the prevalence and severity of the pathological conditions present.

## Discussion

The isotopic and palaeopathological evidence presented above creates a convincing case for these children being pauper apprentices, with many likely indentured and transported from London. Not only were their isotope profiles indicative of a non-local origin, their diet was also distinctive in terms of low animal protein content compared to the named individuals. This study has provided a rare opportunity to make a direct comparison between these children and individuals of known provenance. All the different strands of data distinguish these children not only as ‘other’, but also ‘lesser’. Their struggles and their deprivation are written on their skeletons. The palaeopathological data, including evidence of childhood adversity such as rickets and enamel hypoplasia, point unequivocally to childhood deprivation. This adversity was not of short duration: the hypoplastic lesions are extensive and present on tooth crowns forming at different ages, including deciduous teeth, indicating poor intrauterine health which continued postnatally. The evidence for very high levels of respiratory disease, a known occupational hazard of working in textile factories, further supports the idea that these children were mill-workers. When all this evidence is integrated with the rich historical data, including the indentures of children sent from urban workhouses to West House mill and the burial records, then the evidence is overwhelming. This is the first time that direct skeletal testimonies of the lives of these children have been obtained.

The skeletal evidence for vitamin deficiencies and delayed growth and the carbon and nitrogen isotope data reflect the low animal protein component in the diet. Particularly stark was the similarity between the isotope values from these children and those from the Kilkenny Workhouse assemblage, which relate to the period of the Great Famine in Ireland and a period of extreme malnourishment and starvation. Historical studies emphasize the limited and low-protein content of the provisions available to child workers and this is evidenced in the skeletal remains [7, 15]. Given that some of children buried at Fewston are known to have died within a few years of arrival, their preceding workhouse diet is also relevant for interpreting these isotope ratios, providing a damning picture of the care of children in urban workhouses in England.

**Fig 11:**
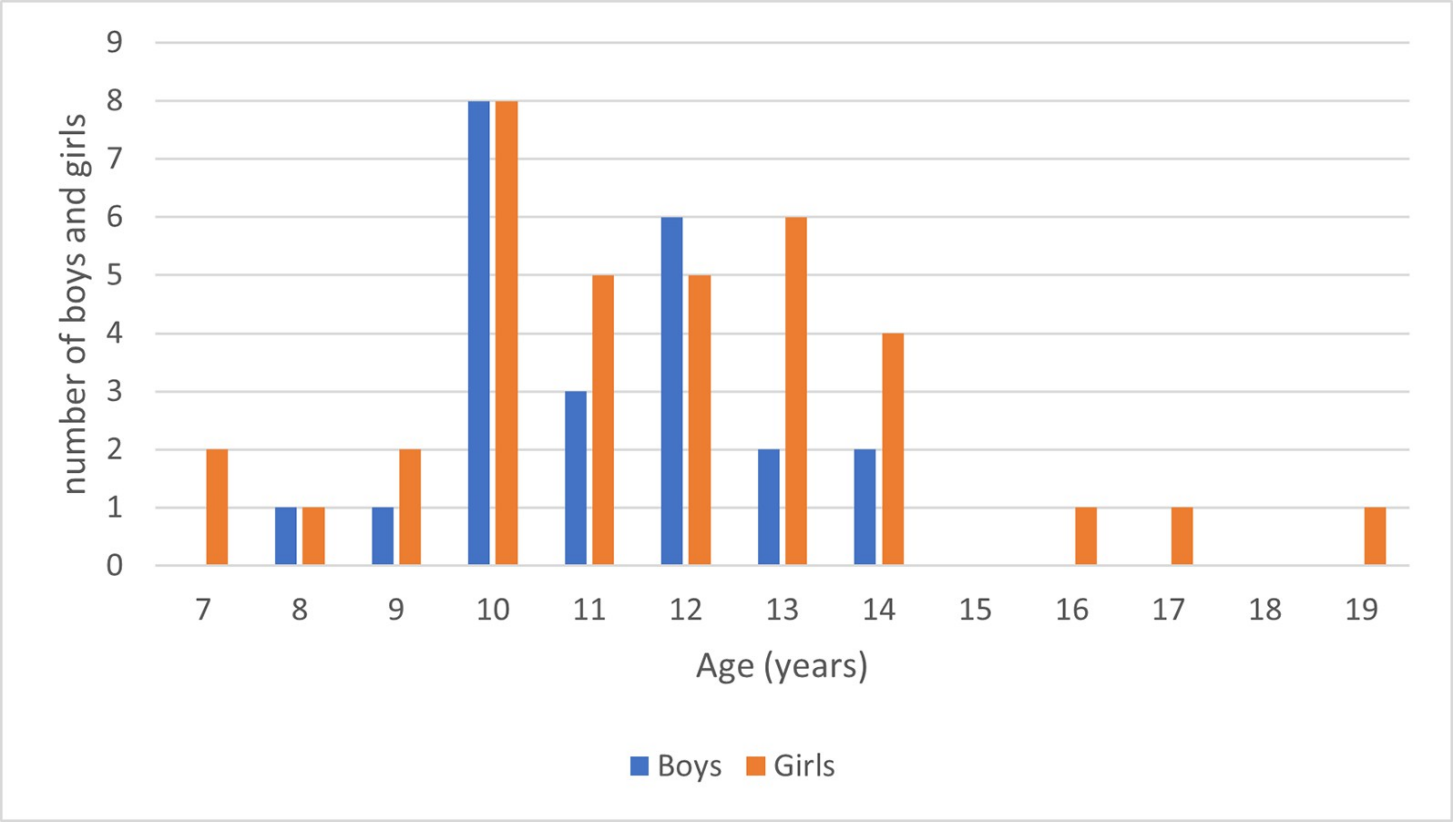
Age distribution of boys and girls known to have been sent to Fewston parish as pauper apprentices.

The ages at which the children were indentured from the workhouse to the Fewston parish is significant in terms of interpreting the pathological conditions, many of which point to early childhood poverty rather than directly to the working conditions in West House mill. Humphries [15] and Honeyman [7] have highlighted that the average age of indenture was often around 11–12 years, although child workers under 10 years were not uncommon. A summary of the age distribution of the children apprenticed to the Fewston mill is provided in Fig 11.

Robert Blincoe (discussed above) was a contemporary of some of the Fewston apprentices and was likewise indentured from a London workhouse (St Pancras) to mills in the North (Nottinghamshire and Derbyshire) from the age of 7 years. A key motivation for Robert when agreeing to sign the indenture was the promise of better food: the children were told that “they would be fed on roast beef and plum pudding”–an enticing prospect for a workhouse child accustomed to a meagre and monotonous diet [14]. Robert soon laments the reality of his daily diet, which consisted almost entirely of porridge and black bread. Such a diet is not only nutritionally limited but is unlikely to supply the necessary energetic demands for growing children to function optimally during a 14-hour working day. It is unsurprising, therefore, that many of the children showed delayed growth into the late teens, given the necessary life-history trade-offs between growth and immune function.

By contrast, the named local individuals at Fewston had occupations that included farming and skilled work (e.g., tailor, stone mason). The relative wealth of these people in this small rural community meant that they consumed a better-quality diet. The remarkable diaries of the 19^th^ century Fewston resident John Dickinson (son of Mary and John Dickenson, SK310 and SK351 sampled here) indicates that pig and cattle were commonly consumed by those within the social sphere of the named individuals. This is evidenced in both the carbon and nitrogen isotope values and the lower prevalence of pathological lesions associated with childhood adversity (e.g. enamel hypoplasia) in the named individuals. It is important to note, however, that not all families in the vicinity of Fewston were as comfortably situated; rural poverty was stark in many parts of the country during the 18^th^ and 19^th^ centuries. This is reflected in the range of isotopic values revealed by the ‘unknown’ adult group, which also indicates impoverished diets at the lower end of the scale. Robert Collyer, who worked in the Fewston mill as a child, was not a pauper apprentice (although both his parents had been), but recalls his childhood diet as comprising “oatmeal, and what we call mush…., and skim-milk….Not much meat, for meat was dear, but soup with dumplings” [26 (p32)].

During the last quarter of the 19^th^ century, Fewston was also known to be home to itinerant laborers (referred to as ‘navvies’ or ‘men on the tramp’) who were involved in the construction of the nearby reservoirs. There are historical accounts of the poor conditions and hunger experienced by such workers. For example, Elizabeth Garnett [107] published a letter in the *Leeds Mercury* newspaper on the 25th of February 1879 detailing the condition of a small number of navvy families: “woman, with three children, living in a cow-house, no food; two elder boys, aged 13 and 15 (navvy lads) out of work, nothing to eat”… “navvy and wife, living in one room: starving”… “engine-man (out of work), wife ill fifteen years. They had no food”.

Working conditions within the factories and the care and treatment of apprentices varied widely between mills. Prior to the Factory Act of 1833, children were often required to work 14 hours a day, as described in Robert Collyer’s biography [28]. In return for their labour, the masters housed and fed the children, gave them some rudimentary education and they were taught a trade (albeit a fairly unskilled one). Collyer’s account of working in West House Mill also attests to the commonplace use of corporal punishment–children were regularly beaten with a leather strap [28 (p15)]. Some textile enterprises were notorious for their mistreatment of parish apprentices and Colbeck and Company, the owners of West House mill, had a reputation for brutal working conditions. A farmer in the Fewston parish, Moses French, wrote a poem in 1877 entitled ‘Mr Colbeck’s Spinning Mill’ “*… hundreds he employed/And in his service many a life destroyed… Some they maimed or killed outright/ By running them both day and night”* (as cited in Fackrell and Hart [27 (p127)]). Moses was aged 76 at the time of writing the poem and so would have been a contemporary of some of the pauper apprentices working at the mill during the early 19^th^ century. It is worth noting, however, an inspection of the mill in 1802 by officials for the ‘Health and Morals Apprentices Act’ found: no evidence of ill treatment, that the mill was well ventilated, the children were not required to work during the night, and they were provided with new clothes each year [27].

In addition to corporal punishment, the work was dangerous for tired and malnourished children who were at risk falling asleep adjacent to heavy machinery that was in perpetual motion. Robert Blincoe witnessed several of his peers being horrifically injured through working with heavy machinery and lost several fingers himself in a work-place accident [14]. Industrial injuries went largely un-reported until the enactment of safety legislation in 1844 [16]. One account of an accident in one of the Fewston cotton mills was recorded in 1866: Henry Ludley Marwood, a local child apprenticed to the mill, caught his arm in a carding machine, which caused extensive soft tissue damage. He was subsequently taken to Leeds infirmary but died a week later whilst having his arm amputated [27]. Returning to the present, we see similar risks of trauma today: the International Labour Organizations’ report on hazardous child labour, noted a high number of traumatic injuries amongst adolescent child workers, and it is estimated that currently 22,000 children are killed at work every year [108, 109]. In addition, the physical abuse of children persists today in manufacturing industries, affecting at least 7% of those engaged in this work [108]. Amongst the Fewston non-adults there is limited evidence of traumatic injuries, although analysis is hampered by the generally poor and fragmentary preservation of many of the skeletal remains. One adolescent (SK 271, 17–19 years), however, had two healed fractures in two right ribs and a possible unhealed fracture in a rib shaft fragment. Multiple fractures in different stages of healing are often indicative of abuse, although more evidence is required in this instance for a confident diagnosis.

Some parish workhouses provided a degree of oversight for ‘their’ indentured children, sending representatives to inspect the well-being and working conditions of the children during the apprenticeship period [16]. The parish officers in Hull, for example (who also sent several children to Fewston) made prior investigations before the indentures were signed and considered the long-term career opportunities. However, subsequent inspections of the welfare of children, or communications with employers once apprentices had arrived were infrequent [7, 16]. Effective interventions for the mistreatment of pauper apprentices were rare. A government report noted that out of just over 2,000 paupers apprenticed from London (aged 8–18 years), one-third were not accounted for and at least 4% were known to have died [13].

For these young apprentices, under-nutrition, meagre living conditions, and separation from their family members, combined with long working days, and exposure to toxic and hazardous conditions, had dire consequences for their growing bodies. Flax mills were particularly reliant on the labour of young children whose smaller bodies could more readily crawl under the machinery–to the extent that they were excluded from the earliest factory legislation [16]. The prevalence of rib lesions and maxillary sinusitis in the non-local Fewston sample was higher than observed in a contemporaneous skeletal sample excavated from an urban site, renowned for high levels of industry and air pollution [24]. The especially high prevalence of new bone formation in the upper and lower respiratory tract at Fewston is likely to stem from multiple causes. Maxillary sinusitis and rib lesions can result from numerous factors, including infectious disease, cardiovascular disease, neoplasms, metabolic disease, and particulate pollution [110, 111]. There is, however, a compelling argument that in the isotopically identified non-local children from Fewston it could at least have been exacerbated by the inhalation of cotton particles during mill work. Respiratory conditions, such as byssinosis caused by the inhalation of fibres, were a well-known occupational hazard of textile work [16]. In the 19^th^ century, this condition was sometimes known as cotton-lung, cotton pneumonia, or spinner’s phthisis. Air pollution within the factories was notorious for causing chronic inflammation of both the upper and lower respiratory tracts. In 1861 it was reported that about three quarters of flax-workers had severe bronchial disorders [5]. Similar conditions have been observed in child labourers working in hazardous industries today, especially those which result in the inhalation of particles. Such working conditions have been observed to result in pulmonary difficulties, and additional risks from the toxicity of the particles inhaled, some of which are carcinogenic [108].

One of the named individuals from Fewston, Daniel Fox, is also relevant here. While Daniel was a policeman prior to his early retirement, census records for Selby in Yorkshire indicate that his earlier occupation was a ‘flax dresser’ [90]. Country Tyrone (where he grew up) was a flax growing area and it is very likely that Daniel’s childhood and early adulthood was spent in this industry. An enquiry in 1891–2 noted that 53% of female textile workers in Belfast died from phthisis–far in excess of general mortality from this condition [112]. Daniel died of phthisis at the age of only 33 years. Unfortunately, his ribs were not recovered and could not be examined for skeletal lesions.

At Fewston, severe dental defects in both the deciduous and permanent dentition highlight long-term health deficits. The deciduous teeth begin to develop in utero, whilst the crowns of the permanent teeth form throughout infancy and early childhood. Defects in multiple teeth forming at different ages therefore provide insights into the systemic and chronic nature of the underlying causes of these. The presence of defects in deciduous teeth can be linked to inter-generational health deficits and maternal nutritional deficiencies [113, 114]. If the mother continues to be grossly malnourished, or in poor health after her infant is born, then this can affect the quality and quantity of the milk that she provides to her offspring [115]. This will exacerbate any existing deficiencies experienced by the infant during intrauterine development. The effects of under- and malnutrition at different gestational ages, as well as in early infancy, has been shown to have longer term impacts during later childhood and adulthood [116]. Deprivation through poor nutrition can also result in stunted growth as the body prioritizes other key functions such as the immune system. Such growth delays are not easily overcome within the context of these children’s lives and increased susceptibility to infectious diseases, frailty and lower life expectancy will result [24, 105, 117]. Unfortunately, there is continuing evidence of the impact of child labour on the growth and development of children today. As the International Labour Office [109] highlight, children are more vulnerable to hazardous conditions than adults because they are still growing. One key finding of their research was that the period of physical maturation for children engaged in hazardous labour today often becomes prolonged, sometimes commencing at an earlier age and continuing until the mid-twenties [109].

Given the workhouse origins of the children recovered from Fewston and the low social mobility of this period, it is likely that the children who died in West House mill were born into poverty to mothers whose lives were also spent either destitute or close to it and subsequently malnourished. Inter-generational poverty, prevalent during this period, led to a lack of ‘maternal somatic capital’, ultimately creating what Wells [118] describes as a ‘metabolic ghetto’ and acute health inequalities between the wealthy and the poor. The impact of such a poor start in life would have been compounded by the subsequent working and living conditions upon reaching Fewston, leading to the untimely deaths of some.

Despite the dire circumstances of these children, it is important to consider the power and potential of individual agency to overcome. Robert Blincoe demonstrated this in his several attempts to escape the mills to which he was sent (alas he was discovered and returned) and in his subsequent relative success in later life, which was in part due to some rudimentary education and skills developed during his apprenticeship [14]. Educational provision by mill owners in the form of Sunday Schools likely furnished children with key literacy skills that would have been valuable to their post-apprenticeship employment prospects. In 1815, records show a private Sunday School for the instruction of 500 pauper apprentices bound to West House mill, despite such provision not being compulsory until 1833 [27]. We know that Robert Collyer’s parents were both pauper apprentices in West House mill, and while they continued in a state of relative poverty post-apprenticeship (supporting a family of 6 children on 18 shillings a week), necessitating their own son working in the same mill, they nevertheless carved out a life for themselves [28]. Robert eventually emigrated to the USA where he became a prominent Methodist minister in Chicago. Mary Burgess, who was apprenticed to West House mill from St George the Martyr Parish in Southwark, London at the age of 10 years, survived her apprenticeship, married a weaver, had 6 children, moved to Leeds, then Barnsley, and in later life was comfortably situated enough to employ two domestic servants [27]. Some scattered stories of survival and success emerge from the grime; nevertheless, these are few when compared to the tribulations, illness and death faced by the many.

## Conclusion

This study provides a direct and compelling testimony of the impact of poverty and factory labour on children’s growth, health and mortality in the past. Unfortunately, the deplorable lives of these children are analogous to those engaged in hazardous labour today. This is the first bioarchaeological evidence for pauper apprentices in the past and it unequivocally highlights the toll placed on their developing bodies. The children were removed from workhouses and indentured to a life of toil in the mills of the North of England. They were forced to sacrifice themselves to conditions that were detrimental to their physical and mental wellbeing, in some instances leading to their death within a few years of apprenticeship. The children and their families (if alive) were given little choice but to participate in this system of structural violence. The very low life expectancy of the labouring classes during this period has been well documented by historians and dramatised in novels, and it is therefore easy to become inured to tales of such hardships. However, the story of the pauper children, taken from workhouses to provide grist to the northern mills provides a particularly acute glimpse of just how denigrated the poor were during this period. It also provides stark physical evidence of the degradations faced by child labourers engaged in similarly hazardous work around the world today.

The skeletal analysis from Fewston immediately marked these children as distinctive due to the anomalous numbers of adolescents, the severity of the skeletal markers of adversity, and high prevalence of respiratory disease. We now know that these skeletal indicators of disease relate to an impoverished urban childhood, leading to compromised physiologies. This frailty was then compounded by long hours of work in a polluted atmosphere, amidst noisy and dangerous machinery, and with a lack of adequate rest and diet. These children, far away from home, were provided with little or no protection or oversight from their distant parishes. With no family in close proximity to decry their working conditions, or care if any harm befell them, they were left vulnerable, expendable and forgotten. The evidence from Fewston also highlights the consequences of inter-generational poverty. In the case of many of these children, they were already disadvantaged due to the under-nutrition of their mothers. Poverty was therefore embodied and imprinted upon the physiology of these children prior to birth; the odds were stacked against them even before they drew their first breath. Direct evidence for childhood health in the form of skeletal analysis is essential for providing incontrovertible insights into the lived experiences of these children prior to their untimely deaths. The data presented above provide a unique and compelling testimony of their short lives.

As discussed throughout, this is not a story that rests solely in the past. Today it is estimated that 73 million children (aged between 5–17 years) globally are engaged in hazardous work. This is almost half of the 160 million child labourers [109]. Global efforts to reduce the numbers of children involved in labour, and especially those in hazardous industries, has stalled since COVID [119]. Relatively few studies have documented the impact of such work on the growth and mortality risk of these children. Children in hazardous work have been described as “the silent majority within child labour”, with insufficient interventions [108]. This examination of the skeletons of children engaged in such work in the past highlights the range of issues, including severe growth delay, malnourishment, respiratory disease and low life expectancy.

Whilst hazardous child labour is no longer a key concern for children in the UK, one fifth of children live in poverty, and in the North of England that figure is closer to one third. Steep rises in relative and absolute poverty are expected over the next few years due to the pandemic and the current cost of living crisis. This high level of poverty continues to affect the health and life-long risks of chronic disease, and life expectancy of children today, with consequences that will have inter-generational impacts through poor maternal nutrition [117]. As this study has shown, bioarchaeological analysis can highlight the dire effects of poverty on the bodies of children when such insidious inequalities are allowed to progress unchecked.

## Supporting information

S1 Appendix (DOCX)

S2 Appendix (XLSX)
