# Supplementary material for: The expendables: Bioarchaeological evidence for pauper apprentices in 19th century England and the health consequences of child labour
Source: PLoS One. 2023 May 17;18(5):e0284970. doi: 10.1371/journal.pone.0284970 (PMC10191286; doi:10.1371/journal.pone.0284970)
Supplement: S1 Appendix — (DOCX) [file pone.0284970.s001.docx]

**Supplementary Materials**

**Methods**

**Peptide Analysis**

The peptide extraction and identification methods used for the Fewston assemblage follow the methods as previously described in Stewart et al. (2016, 2017) and Gowland et al. (2021). Initially, the tooth surface was abraded with a dental burr to remove any obvious surface contaminants, after which the enamel was washed with 3% hydrogen peroxide (H_2_O_2_) for 30 seconds before being rinsed with ultrapure water (Elga Purelab Ultra, 18.2 MΩ-cm). The cap of a 0.2 millilitres (mL) microcentrifuge tube (Eppendorf) was filled with approximately 60 microliters (µL) of 5% (vol/vol) hydrochloric acid (HCl). The tooth was lowered onto the HCl solution with contact maintained for 2 minutes to complete the initial etch. The first etch was discarded and the process was repeated for a second time with the second etch retained as the etch solution.  Using a pipette set to 10 µL, a C18 resin loaded ZipTip™ (ZTC18S096; EMD Millipore) was conditioned three times with 100% acetonitrile, followed by three times with 0.1% (vol/vol) formic acid; each draw of acetonitrile and formic acid was discarded. After the ZipTip™ was conditioned, the etch solution was bound to the ZipTip™ by drawing the etch solution through the ZipTip™ 10 times, discarding the last draw. The ZipTip™ was then washed six times with 0.1% (vol/vol) formic acid, with the solution from each wash discarded. The resin-bound peptides were eluted from the ZipTip™ by drawing a 4 µL 60% acetonitrile/0.1% formic acid elution buffer through the ZipTip™ 10 times. The eluted peptides were subsequently freeze-dried to ensure stability until they could be analysed.

The freeze-dried samples were dissolved in 12 µL of 0.1% trifluoroacetic acid (TFA) in water, centrifuged for 5 minutes on a desktop centrifuge, and 10 µL transferred to glass autosampler vials. A sample of 5 µL was then injected for analysis by reversed-phase nano-flow liquid chromatography tandem mass spectrometry (nanoLC-MS) (UltiMate 3000 RSLCnano; Thermo Fisher Scientific) coupled to a hybrid quadrupole Orbitrap mass spectrometer (Q Exactive Orbitrap; Thermo Fisher Scientific) equipped with a nanospray ion source (Nanospray Flex, Thermo Fisher Scientific). Peptides were first loaded onto a C18 trapping cartridge (Pepmap100 C18; Thermo Fisher Scientific; 0.3 × 5 millimeter [mm] i.d.; 5 micrometer [μm] particle size) for 4 minutes at a flow rate of 20 µL/min using mobile phase A (0.1% (vol/vol) formic acid in hypergrade water, Merck KGaA). Separation was achieved at a flow rate of 200 nanolitre (nL)/min on an analytical column (PepMap100 C18; 15 centimetre (cm) × 75 μm; 2 μm particle size) using a gradient of mobile phase B (0.1% (vol/vol) formic acid in acetonitrile, LiChrosolv, Merck KGaA) from 1% to 28% B (curve 4) over 42 minutes, 28% to 99% B (curve 6) over 8 minutes, 99% B for 5 minutes and back to 1% B over 1 minute to equilibrate for 9 minutes, with a total chromatographic run time of 65 minutes. A stainless-steel emitter (40 mm, 1/32” OD) was used post column. Blank injections were used between runs to reduce possible carryover. The mass spectrometer was operated in the positive ion mode with a spray voltage 1.6 kV and a capillary temperature of 250°C. Mass Spectrometry data was acquired in a data dependent manner; with full scan MS spectra (300-1,650 m/z, (R = 140,000 at 200 *m/z*) followed by the fragmentation of the top 10 most abundant precursor ions. A lock mass of 445.1200 *m/z*, corresponding to polysiloxane ([M+H]^+^, (C_2_H_6_SiO)_6_), was used. Dynamic exclusion was set to 45 seconds (s), with charge exclusion set for unassigned and singly charged species. Automatic gain control (AGC) target was set to 1 x 10^6^ with a maximum injection time of 20 milliseconds (ms) for full scan. Fragmentation of precursor ions was performed by higher-energy collisional dissociation (HCD) with a normalized stepped collision energy of 20, 25 and 30, with a default charge state of 2. MS/MS scans (R = 17,500 at 200 *m/z*) were performed with an AGC target value of 1 x 10^5^ and a maximum injection time of 120 ms using an isolation window of 2.2 *m/z*. An inclusion list of 440.2233 *m/z* and 540.2796 *m/z*; corresponding to Ser-Met(oxidised)-Ile-Arg-Pro-Pro-Tyr (from AMELY) and Ser-Ile-Arg-Pro-Pro-Tyr-Pro-Ser-Tyr (from AMELX), respectively, was used. Sex was determined by visualising the reconstructed ion chromatogram of 440.2233 *m/z* and 540.2796 *m/z* at 1 PPM mass accuracy; corresponding to Ser-Met(oxidised)-Ile-Arg-Pro-Pro-Tyr (from AMELY) and Ser-Ile-Arg-Pro-Pro-Tyr-Pro-Ser-Tyr (from AMELX), respectively. Determination was further supported with correct relative retention times, a correct charge state of 2 in the full MS and an accompanying MS/MS spectrum matching predicted fragment ions.

**Results**

**Strontium and Oxygen Analysis**

**Supplementary Table 1: Strontium and Oxygen Isotope Results.**

| **Sk #** | **Name** | **Age** | **Sex** | **Tooth^a^** | **Sample Weight (Sr)** | **87Sr/^86^Sr** | **Sr 2 SE** | **Sample Weight (O)** | **𝛿18Oc ‰** | **𝛿18Op‰** |
| --- | --- | --- | --- | --- | --- | --- | --- | --- | --- | --- |
| SK056 |  | 7.5-8.5 | F | URM2 | 16.3 | 0.713483 | 0.000010 | 15.51 | 26.5 | 17.7 |
| SK071 |  | 7-9 | U | LLM2 | 15.9 | 0.710388 | 0.000011 | 13.29 | 25.3 | 16.5 |
| SK077 |  | 17-20 | M? | URM2 | 13.9 | 0.713538 | 0.000013 | 16.92 | 26.3 | 17.4 |
| SK116 |  | 11-13 | F | URP2 | 22.1 | 0.709784 | 0.000013 | 16.4 | 25.1 | 16.2 |
| SK208 |  | 13-14 | F | URP2 | 17.9 | 0.709143 | 0.000013 | 9.8 | 25.4 | 16.5 |
| SK214 |  | 17-19 | U | LRP2 | 13.4 | 0.709318 | 0.000011 | 14.82 | 24.8 | 15.9 |
| SK217 |  | 14-16 | U | LLM2 | 25 | 0.709856 | 0.000013 | 12.09 | 25.8 | 16.9 |
| SK223 |  | 14-15 | M | ULP2 | 14.3 | 0.709459 | 0.000010 | 12.81 | 25.6 | 16.7 |
| SK229 |  | 14-16.5 | U | LRP2 | 17.7 | 0.709108 | 0.000015 | 17.42 | 25.7 | 16.8 |
| SK232 |  | 9.5-11 | U | ULP2 | 21.6 | 0.70885 | 0.000014 | 12.43 | 26.1 | 17.3 |
| SK250 |  | 10.5-12.5 | F | LLM2 | 14.6 | 0.709205 | 0.000009 | 15.28 | 25.7 | 16.8 |
| SK262 |  | 16-18 | U | ULP2 | 14.2 | 0.711331 | 0.000008 | 13.93 | 25.1 | 16.2 |
| SK265 |  | 18? | U | LLM2 | 18.4 | 0.709227 | 0.000010 | 13.49 | 25.4 | 16.6 |
| SK331 |  | 14-16 | M | LLP2 | 13.8 | 0.709839 | 0.000010 | 17.51 | 25.0 | 16.2 |
| SK334 |  | 11-13 | M | LLP2 | 12.5 | 0.709059 | 0.000010 | 17.24 | 25.4 | 16.5 |
| SK338 |  | 11-14 | M | ULP2 | 15.8 | 0.709318 | 0.000011 | 19.78 | 26.1 | 17.3 |
| SK348 |  | 17-25 | F | ULP2 | 14.1 | 0.70904 | 0.000012 | 13.68 | 25.4 | 16.5 |
| SK423 |  | 13-14 | F | LLP2 | 18.9 | 0.713262 | 0.000012 | 12.09 | 25.6 | 16.7 |
| SK438 |  | 15-18 | F | LRP2 | 12.5 | 0.709208 | 0.000014 |  | - | - |
| SK119 | Matthew Marjerrison | 38 | M | LRP2 | 14.2 | 0.710254 | 0.000018 | 16.4 | 25.2 | 16.4 |
| SK130 | George Lister | 66 | M | ULP2 | 21.3 | 0.710704 | 0.000014 | 14.2 | 25.3 | 16.5 |
| SK226 | David Lister | 84 | M | LLP2 | 15.2 | 0.711133 | 0.000021 | 10.7 | 25.3 | 16.4 |
| SK342 | Bentley Darnbrook | 26 | M | LRP2 | 18 | 0.711462 | 0.000013 | 12.09 | 24.8 | 15.9 |
| SK319 | Sarah Darnbrook | 22 | F | URP2 | 14.3 | 0.710961 | 0.000013 | 18.05 | 24.8 | 15.9 |
| SK351 | John Dickinson | 63 | M | LRP2 | 12.4 | 0.711243 | 0.000015 | 16.8 | 25.4 | 16.5 |
| SK363 | Sarah Gill | 54 | F | ULC | 17.9 | 0.71039 | 0.000010 | 10.7 | 25.2 | 16.3 |
| SK339 | Richard Gill | 41 | M | LLP2 | 11.4 | 0.710186 | 0.000014 | 12.5 | 25.0 | 16.1 |
| SK366 | John Renton Newsome | 76 | M | LRP2 | 17 | 0.710897 | 0.000016 | 10.7 | 24.8 | 15.9 |
| SK378 | Eliza Wigglesworth | 34 | F | URM2 | 20.1 | 0.710317 | 0.000015 | 17.7 | 25.4 | 16.5 |
| SK441 | Daniel Fox | 33 | M | ULP2 | 18.6 | 0.708845 | 0.000012 | 12.4 | 24.7 | 15.8 |
| SK088 | Unknown | 38 | M | LLP2 | 20.1 | 0.712592 | 0.000011 | 12.47 | 25.3 | 16.4 |

^a^ U=upper or maxillary, L=lower or mandibular, R=right, L=left, C=canine, P=premolar, M=molar, and number indicates position
